# Patient experiences with general practitioners: psychometric performance of the generic PEQ-GP instrument among patients with chronic conditions

**DOI:** 10.1093/fampra/cmab133

**Published:** 2021-10-20

**Authors:** Øyvind A Bjertnæs, Hilde H Iversen, Jose M Valderas

**Affiliations:** 1 Department of Health Services Research, Division for Health Services, Norwegian Institute of Public Health, Oslo, Norway; 2 Health Services and Policy Research Group, Exeter Collaboration for Academic Primary Care, University of Exeter Medical School, Exeter, United Kingdom

**Keywords:** general practice, general practitioner, health care surveys, patient satisfaction, physician–patient relations, psychometrics

## Abstract

**Background:**

Most generic patient experience instruments have not been validated specifically for persons with chronic health problems, even though they are the dominant user of GPs/family physicians.

**Objectives:**

To assess the psychometric properties of the generic Patient Experiences with GP Questionnaire (PEQ-GP) instrument (five scales: assessment of GP, coordination, patient enablement, accessibility, and practice) in persons with chronic conditions, and to develop a short version to maximize response rates and minimize respondent fatigue in future applications.

**Methods:**

Secondary analysis of data from a national survey of patient experiences with general practitioners in 2018–2019 (response rate: 42.6%). The psychometric properties of PEQ-GP were assessed with exploratory factor analysis and Cronbach’s alpha, supplemented with confirmatory factor analysis (CFA) and item response theory (IRT). A short version was constructed and evaluated based on item performance.

**Results:**

Nine hundred and seventy persons reported a chronic condition(s), the most frequent being “musculoskeletal, arthritis, other back and joints” (*n* = 473, 48.8%). Factor analysis identified three scales with adequate psychometric results: GP (15 items; Cronbach’s alpha: 0.96), practice (3 items; Cronbach’s alpha: 0.87), and accessibility (2 items; Cronbach’s alpha: 0.77). Evaluation of item performance identified a 7-item short version, including a 5-item GP scale with scores with strong concordance with the 15-item scale (Intraclass Correlation Coefficient: 0.97, *P* < 0.001).

**Conclusions:**

The generic PEQ-GP exhibits adequate psychometric performance for persons with chronic conditions. Three empirically derived PEQ-GP scales cover evaluation of the GP, accessibility, and practice. The 7-item short form minimize respondent burden, but further validation work is warranted before large-scale use.

**Key Messages**

- The PEQ-GP exhibited adequate performance for persons with chronic conditions.
- The PEQ-GP cover three scales for evaluating the GP, accessibility, and practice.
- A 7-item short form was identified that minimize respondent burden.

## Introduction

Positive patient experiences and high patient satisfaction are important outcomes of high-quality health care,^1^ and are associated with better adherence, clinical outcomes and patient safety, and less health care utilization.^2–4^ The measurement of patient experiences is often based on surveys, with a majority of national and cross-national surveys conducted among patients in secondary health care.^5^ However, several large-scale initiatives exist for primary care practices or general practice including the General Practitioner (GP) Patient Survey in United Kingdom,^6^ the Consumer Assessment of Healthcare Providers and Systems (CAHPS) Clinician and Group Survey in the United States,^7^ the EUROPEP instrument,^8,9^ the Person-Centered Primary Care Measure,^10^ and the Primary Care Assessment Tools.^11^ A systematic review of patient-based instruments for the assessment of individual physician performance showed that all used a generic questionnaire, but found large variation in the underlying conceptual model, data collection procedures and questionnaire content.^12^

Most patient experience instruments with GPs/family physicians are generic, implying that the topics included should be relevant and important for most patients. A dominant user of GPs/family physicians are persons with chronic health problems, e.g. a British study found that 80% of all GP consultations were with persons with chronic health problems.^13^ The health and health care needs of such persons are different than those without chronic problems, e.g. the first are more dependent on multiple health services and need to learn how to self-manage and cope with their health problems. Given the different needs and use of services, the question arises as to how valid generic instruments of patients’ experiences with their physician are when used to measure and report the experiences of people with chronic problems. Most generic patient experience instruments have not been validated specifically for persons with chronic health problems, as can be seen in the review of patient-based instruments for assessing physician performance.^12^

The Norwegian Patient Experiences with GP Questionnaire (PEQ-GP) consists of five scales: assessment of the GP, coordination, patient enablement, accessibility and practice.^14^ The PEQ-GP was developed without specific consideration to the context and needs of persons with chronic conditions. Evidence for its performance is therefore required for applications requiring a specific chronic care approach, such as the evaluation of different health services involved in chronic care, integration of care and more in-depth evaluations of coordination.^15,16^ Furthermore, the use of instruments in clinical settings may benefit from minimization of the administration burden, as to maximizing response rates and reducing respondent fatigue. The main objective of this study was to assess the psychometric properties of the PEQ-GP instrument for persons with chronic conditions. The secondary objective was to identify a parsimonious set of PEQ-GP items and develop a short form for persons with chronic conditions. The study used nationally representative data for PEQ-GP in Norway and has relevance for all other countries with similar organization of care.

## Methods

### Setting

All residents in Norway are entitled to a regular GP, and more than 99% of the population are on a regular GP’s patient list.^17^ The GPs have a medical professional and coordination role and are required to collaborate with other services about their list patients, including persons with chronic conditions.

### Data

This was a secondary analysis of data from a national survey of patient experiences with general practice in 2018–2019. The data collection has been described elsewhere.^18^ The total patient sample was 5,000, of which 2,029 responded (42.6%). Background data about the patients were obtained from public registries. In this study, we selected respondents 45 years and older with one or more self-reported long-lasting conditions (defined as lasting 6 months or longer, or new conditions expected to be long-lasting), and who had been in contact with the GP the last year.

### Questionnaire and scales

The generic PEQ-GP consists of five scales with 18 items^14^: assessment of GP (eight items), coordination (two items), patient enablement (three items), accessibility (two items) and practice (three items). The questionnaire also consists of three additional single items, and two overall health care experience items (general satisfaction, recommendation of the GP to friends/family). All items have a 5-point response format ranging from 1 (“not at all”) to 5 (“to a very large extent”), with the additional response “not applicable.”

The conceptual model of quality is based on OECD’s quality indicator project, operationalizing patient safety, effective treatment and patient centredness as the core dimensions of quality.^19,20^ While patients are broadly involved in the measurement of quality, the patient centredness component is measured by patient-reported experiences of care. The latter is defined as individual perceptions on the degree to which health service delivery respond to individual needs, in line with the definition proposed in OECD’s Patient-Reported Indicator survey (PaRIS). We built on the scales from the generic PEQ-GP, but included enablement and coordination in the GP factor. All questions about enablement and coordination include a direct assessment of the GP, in line with the other GP questions, and have high relevance for patients with chronic conditions. In contrast, enablement and coordination are not relevant for many patients without chronic conditions, which is why they were treated as separate factors in the generic PEQ-GP. Thus, the conceptual model we proposed for chronic patients consisted of three first order factors of GP experience: accessibility, practice, and GP. In accordance with previous research,^9,21^ patients’ assessment of their GP can be expected to constitute a strong unidimensional construct containing all the various aspects of GP performance. We thus also hypothesized a second order factor labelled “patient-perceived quality of general practice.” The second order factor would explain the associations between first order factors (i.e. all relate to perceived quality of general practice), but each of the first order factors would be separate latent constructs with their own manifest indicators.

### Statistical analysis

Items were assessed for missing data and ceiling effects. Exploratory factor analysis was used to assess the underlying structure of the items (principal axis factoring, Promax rotation, factors with eigenvalue above 1), while internal consistency reliability was used to assess if items adequately contribute to the scale construct (item-total correlation, Cronbach’s alpha, Cronbach’s alpha if item deleted).

We subsequently conducted confirmatory factor analysis to verify the factor structure, and item response theory to further evaluate item performance. Full information maximum likelihood (FIML) was used to account for missing data in the confirmatory factor analysis, with estimation that uses all cases including cases with missing on one or several items.^22^ There is no consensus in the literature on the cut-off values for good fit in CFA. Thus, we inspected a range of fit indexes, including the root mean square error of approximation (RMSEA), standardized root mean squared residual (SRMR), the goodness-of-fit index (GFI), the comparative fit index (CFI) and the incremental fit index (IFI). Based on a previous study we considered RMSEA of 0.05 or less, and a GFI and CFI of 0.90 or above to indicate a good fit.^23^ The IFI values range from 0 to 1, with larger values indicating a better goodness of fit, while the SRMR values range between 0 and 1, with 0 indicating a perfect fit. We applied the graded response model for polytomous items in item response theory, and evaluated item performance in terms of item discrimination, item category location (i.e. difficulty) and item fit.^22,24^

Items for the development of a shorter version of the instrument were identified by assessing item missingness,^9^ ceiling effects,^25^ factor loadings in CFA and item performance in the IRT analysis, and as stratified by core aspects to secure content coverage. The IRT analysis was only conducted on the GP scale, since the small number of items (three and two) in the other scales made them less suitable for IRT. The five core aspects of the GP scale were GP patient centredness, GP information, GP coordination, GP enablement and GP overall assessment. Item performance in IRT was based on an assessment of discrimination (higher means better) and difficulty (threshold separation for scale coverage (Wright map), in addition to the *S*−*χ*^2^ statistic. For the latter, a significant result indicates poor item fit.^24^ We selected the best performing item for each of the five GP aspects, and one item from each of the other scales (accessibility, practice). Thus, we selected seven items for the short form. The concordance between the long and short version of the GP scale was assessed using the Intraclass Correlation Coefficient (ICC).

Descriptives, exploratory factor analysis and reliability tests were conducted with SPSS26.0, while confirmatory factor analysis and item response theory analysis were conducted using R version 3.6.3 (packages lavaan, semPlot, mirt, WrightMap).

## Results

Nine hundred and seventy patients were 45 years or older, reported one or more chronic conditions, and had at least one contact with the GP the last 12 months. Of these, 55.7% were females, 80.5% were in the age interval 50–79, and 41.3% had a university degree (Table 1). The most frequent conditions were “musculoskeletal, arthritis, other back and joints” (*n* = 473, 48.8%), “high blood pressure” (*n* = 361, 37.2%), and the umbrella category “other chronic conditions” (*n* = 319, 32.9%). More than half of the respondents (53.2%) reported two or more chronic conditions.

**Table 1.** Descriptives for respondents with chronic conditions (*n* = 970).

	Frequency	%
Gender		
Male	430	44.3
Female	540	55.7
Age		
45–49	79	8.1
50–66	440	45.4
67–79	340	35.1
80+	111	11.5
Education		
Primary school	183	19.3
High school	372	39.3
University, <4 years	235	24.8
University, 4 years or more	156	16.5
Number of chronic conditions^a^		
1	454	46.8
2	311	32.1
3+	205	21.1
Type of chronic condition^a^		
Musculoskeletal, arthritis, other back and joints	473	48.8
High blood pressure	361	37.2
Other	319	32.9
Heart condition, incl. myocardial infarction	151	15.6
Asthma, COPD, other breathing	149	15.4
Depression, anxiety, other mental	125	12.9
Diabetes	121	12.5
Cancer	75	7.7
Had stroke	44	4.5
Addiction	9	0.9
Self-perceived health		
Very poor	20	2.1
Rather poor	79	8.2
Both poor and good	335	34.6
Rather good	462	47.7
Very good	73	7.5
Number of GP contacts last 12 months		
1	100	10.4
2–5	607	63.2
6–12	209	21.7
13+	45	4.7

^a^Patients could tick all relevant chronic conditions.

**Fig. 1.**
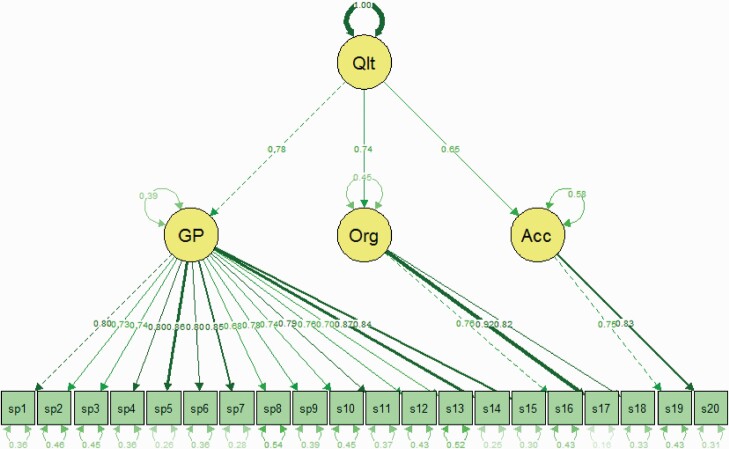
Confirmatory factor analysis model for PEQ-GP for persons with chronic conditions.

The percentage of missing or not applicable ranged from 0.6% for item 14 (overall satisfaction) to 25.9% for item 10 (cooperation with other services), while the ceiling effect varied from 14.7% for item 13 (contact with GP better helped to stay healthy) to 48.8% for item 3 about GP talking in a way the patient would understand (Table 2). Exploratory factor analysis identified three factors with an eigenvalue above 1, explaining 70.3% of the variation of the observed variables (Table 3). The first factor consisted of 15 items related to the GP, the second one of three items on organization and auxiliary staff, and the third one of two items on accessibility. Satisfactory internal consistency was observed for the three scales, with Cronbach’s alpha values of 0.960, 0.868 and 0.774, respectively (Table 2). Confirmatory factor analysis (Fig. 1) showed a reasonable model fit to the data for the three-factor solution (*χ*^2^ = 1439.02, *P* < 0.001, df = 167, RMSEA = 0.089, SRMR = 0.046, GFI = 0.98, CFI = 0.91, IFI = 0.91).

**Table 2.** Psychometric properties of the revised PEQ-GP scales (GP, practice, and accessibility): missing, ceiling effects and internal consistency reliability.

	Missing/not applicable (%)	Ceiling (%)	Item-total correlation	Cronbach’s alpha	Cronbach’s alpha if item deleted
GP			–	0.960	–
1. GP takes you seriously	51 (5.3)	445 (45.9)	0.789	–	0.957
2. GP spends enough time with you^a^	52 (5.4)	274 (29.8)	0.715	–	0.959
3. GP talks to you in a way you understand	56 (5.8)	446 (48.8)	0.690	–	0.959
4. GP is professionally competent	59 (6.1)	378 (39.0)	0.779	–	0.958
5. GP shows interest in your situation	57 (5.9)	365 (40.0)	0.842	–	0.956
6. GP includes you as much as you would like in decisions concerning you	80 (8.2)	323 (36.3)	0.785	–	0.957
7. GP provide sufficient information about health problems and treatment^a^	70 (7.2)	286 (29.5)	0.843	–	0.956
8. GP provide sufficient information about use/side effects of medication	112 (12.5)	162 (18.9)	0.689	–	0.960
9. GP is good at coordinating the range of health services available to you^a^	217 (22.4)	220 (29.2)	0.772	–	0.957
10. GP cooperates well with other services you need	251 (25.9)	209 (29.1)	0.740	–	0.958
11. Contact with GP make you better able to understand your health problems^a^	58 (6.0)	183 (20.1)	0.806	–	0.957
12. Contact with GP make you better able to cope with your health problems	75 (7.7)	140 (15.6)	0.788	–	0.957
13. Contact with GP better help you to stay healthy	88 (9.1)	130 (14.7)	0.722	–	0.959
14. Overall satisfaction with GP^a^	6 (0.6)	461 (47.8)	0.851	–	0.956
15. Recommend GP to family/friends	74 (7.6)	341 (38.1)	0.821	–	0.957
Practice				0.868	
16. GP practice well organized^a^	66 (6.8)	270 (29.9)	0.681	–	0.875
17. Other employees helpful and competent	61 (6.3)	362 (39.8)	0.813	–	0.754
18. Treated with courtesy and respect at the reception	58 (6.0)	415 (45.5)	0.752	–	0.809
Accessibility				0.774	
19. Waiting time for your last urgent appointment acceptable	147 (15.2)	285 (34.6)	0.631	–	–
20. Waiting time for appointments that are not urgent acceptable^a^	83 (8.6)	163 (18.4)	0.631	–	–

^a^Items finally selected for the short form.

**Table 3.** Exploratory factor analysis with loadings^a^ (*n* = 476).

	Factor 1	Factor 2	Factor 3
Factors/items			
GP			
1. GP takes you seriously	0.804		
2. GP spends enough time with you	0.644		
3. GP talks to you in a way you understand	0.656		
4. GP is professionally competent	0.831		
5. GP shows interest in your situation	0.860		
6. GP includes you as much as you would like in decisions concerning you	0.772		
7. GP provide sufficient information about health problems and treatment	0.878		
8. GP provide sufficient information about use/side effects of medication	0.660		
9. GP is good at coordinating the range of health services available to you	0.748		
10. GP cooperates well with other services you need	0.714		
11. Contact with GP make you better able to understand your health problems	0.822		
12. Contact with GP make you better able to cope with your health problems	0.817		
13. Contact with GP better help you to stay healthy	0.734		
14. Overall satisfaction with GP	0.909		
15. Recommend GP to family/friends	0.919		
Practice			
16. GP practice well organized		0.638	
17. Other employees helpful and competent		1.000	
18. Treated with courtesy and respect at the reception		0.762	
Accessibility			
19. Waiting time for your last urgent appointment acceptable			0.913
. Waiting time for appointments that are not urgent acceptable			0.689

^a^Values below 0.2 are not shown. Eigenvalues: factor 1: 11.31; factor 2: 1.56; factor 3: 1.19.

IRT analysis of the GP items showed adequate performance for most items (Table 4), except for item 5 “GP shows interest in your situation,” where the test for item fit indicated poor fit (*P* = 0.027). Item discrimination varied from 2.08 for item 8 (GP provide sufficient information about use/side effects of medication) to 5.71 for item 14 (overall satisfaction) (Table 4). Item category thresholds varied across items, but were mostly concentrated below or around the average, indicating that items measure best at the lower and middle end of the scale. The fourth item threshold varied from –0.1 for item 1 (GP takes you seriously) to 1.13 for item 13 (contact with GP better help you to stay healthy). The categorical response curves visualize item discrimination and item category thresholds (Supplementary Material 1), and further shows that the second response category has questionable value for some items (particularly items 1 and 15).

**Table 4.** Parameter estimates from item response theory analysis of the revised PEQ-GP GP scale (*n* = 567), sorted by core GP aspects^a^.

	*a*	*b*1	*b*2	*b*3	*b*4	*S*-*X*^2^	*P* value
GP patient centredness							
1. GP takes you seriously	3.87	−2.69	−2.29	−1.40	−0.10	28.82	0.422
2. GP spends enough time with you	2.36	−2.84	−1.88	−0.93	0.54	47.82	0.359
3. GP talks to you in a way you understand	2.65	−3.19	−2.50	−1.78	−0.00	28.56	0.381
4. GP is professionally competent	3.32	−3.33	−2.48	−1.39	0.10	39.60	0.167
5. GP shows interest in your situation	4.28	−2.71	−1.93	−1.09	0.10	46.63	0.027
6. GP includes you as much as you would like in decisions concerning you	3.25	−2.76	−2.11	−1.10	0.26	41.10	0.16
GP information							
7. GP provide sufficient information about health problems and treatment	4.07	−2.49	−1.83	−0.87	0.37	37.74	0.103
8. GP provide sufficient information about use/side effects of medication	2.08	−2.37	−1.25	−0.34	1.03	59.62	0.346
GP coordination							
9. GP is good at coordinating the range of health services available to you	2.86	−3.27	−2.00	−0.96	0.53	37.63	0.307
10. GP cooperates well with other services you need	2.54	−2.75	−2.00	−0.92	0.57	34.56	0.629
GP enablement							
11. Contact with GP make you better able to understand your health problems	3.21	−2.41	−1.59	−0.59	0.82	28.31	0.816
12. Contact with GP make you better able to cope with your health problems	2.92	−2.33	−1.50	−0.40	0.97	43.10	0.347
13. Contact with GP better help you to stay healthy	2.31	−2.54	−1.50	−0.27	1.13	59.65	0.142
GP overall assessment							
14. Overall satisfaction with GP	5.71	−2.50	−2.02	−1.16	−0.10	20.21	0.629
15. Recommend GP to family/friends	3.69	−1.94	−1.51	−0.73	0.17	45.05	0.306

^a^Graded response model. *a*: discrimination; *b*1–*b*4: thresholds. *S–X*^2^ represents item fit statistics, with *P* values <0.05 indicating lack of fit.

A 7-item short version was identified. Five items from the GP scale was selected after assessing item missing, ceiling effects and psychometric results, sorted by the five core GP aspects to secure content coverage (GP patient centredness, GP information, GP coordination, GP enablement, GP overall assessment). Within the core aspect GP patient centredness, item 2 performed better than the others: “GP spends enough time with you.” The Wright Map confirmed the similarities in thresholds for the patient centredness items (Supplementary Material 2), but also that the item about spending enough time measures at a higher location of the latent construct than the others. This was also the item with lowest ceiling effect (Table 2). For the aspect GP information we selected item 7, because it had less item-missing, stronger connection to the latent factor in CFA and better discrimination in IRT analysis. For GP coordination, GP enablement and GP satisfaction we selected item 9, item 11 and item 14, respectively. The items constituting each of these aspects performed rather similar across the criteria, but we selected these items because they had lower item-missing, slightly higher correlation with the latent factor in CFA and slightly better discrimination in IRT analysis. The last two items in the short-form were “waiting time for non-urgent appointment” (accessibility scale) and “GP practice well organized” (practice scale). The former performed better than the other accessibility items on item-missing, ceiling effect and factor loading in CFA. The latter had substantially lower ceiling effect than the other practice items (Table 2), but scored slightly poorer on the other criteria.

The Intraclass Correlation Coefficient between the 15-item GP scale and the 5-item scale was 0.97 (*P* < 0.001).

## Discussion

The generic PEQ-GP has demonstrated generally strong psychometric performance for persons with chronic conditions, with three empirically based scales covering evaluation of the GP, accessibility and practice. A 7-item short form was identified that minimize respondent burden.

The PEQ-GP was developed as a generic instrument for all adult patients, but also included topics of special relevance for chronic patients including coordination and enablement. This is positive for the content validity of the instrument for chronic patients, and is a prerequisite for considering the use of PEQ-GP by chronic patients. We conceptualized and found empirical support for a second order factor “patient-perceived quality of general practice,” consisting of the three first order factors “accessibility,” “practice” and “GP.” This is somewhat different from other conceptualizations of general practice care, e.g. the original EUROPEP work that included five factors: availability and accessibility, information and support, medical technical care, doctor–patient relationship and organization of services.^26^ Empirical studies have not given support to these five factors,^9,21,27^ but by collapsing the factors two-to-four in EUROPEP into a GP factor it is possible to map the EUROPEP conceptually to the three-factor model in PEQ-GP. Furthermore, the three-factor model concur with the factors in the Consumer Assessment of Healthcare Providers and Systems (CAHPS) Clinician and Group Adult Visit Survey.^7^ All in all, the PEQ-GP has a sound conceptual and empirical foundation for measuring the quality of general practice from the perspective of chronic patients and can be considered as part of the options in the evaluation of different health services involved in chronic care, integration of care and more in-depth evaluations of coordination.

Items in the GP factor showed wide variation in ceiling effects, item discrimination and item category thresholds, contributing to a broad measurement of the GP. However, IRT analysis showed that the reliability was poor at the higher end of the scale, implying that the scale would benefit from some more “difficult” items to try to distinguish better between high scoring patients (those with reporting better experience of care). The inherent positivity bias in satisfaction measurement makes this somewhat challenging,^28^ but should be considered in the further development of the instrument. The lowest scoring aspects in the GP scale relates to enablement and information about medicines, so these are probably the best candidates for constructing more difficult items. A more robust methodological approach could include testing unbalanced response scales to reduce ceiling effects,^29^ but whether or not this also differentiates between current top scoring patients is uncertain. Another approach could be to further utilize free text comments from patients. A previous study showed that almost half of the comments from patients with excellent rating of health services (i.e. top scores) were about negative or mixed experiences,^30^ and automatic sentiment analysis could be used as a tool to create quantitative variables from these comments.^31^ This approach must solve the fact that many patients do not write free text comments. Without a gold standard solution, we believe the best way forward is to test and possibly combine different approaches, like more difficult items, unbalanced response scales and use of free text comments. Regardless of approach, we underline the importance of shorter scales to maximize response rates and minimize respondent fatigue, which also might be facilitated by using a response-adaptive electronic approach.

The two other factors we identified were accessibility and practice, as hypothesized and in line with the original PEQ-GP study. None of the factors are especially strong empirically speaking, at least compared with the GP factor, but their presence concurs with other studies identifying one or several factors at the practice level.^9,21,26,27^ Previous research has shown that accessibility (understood as short waiting times for appointments) is one of the top priorities for patients in general practice,^32^ and accessibility as a separate factor follows most national and international performance frameworks as reviewed by the OECD.^19^ The ceiling effects in our study showed that accessibility, particularly for non-urgent appointments, are one of the largest improvement factors according to chronic patients, giving important clinical support for having this as a separate factor. Furthermore, the national results indicate the special relevance of the practice level for chronic patients: several specific chronic patient groups evaluated the practice better than the GP, and several chronic groups scored the GP significantly lower and the practice level significantly higher than non-chronic groups.^33^ This underlines the importance of the broader health care team for chronic patients, and thus the clinical importance of the practice factor. Both the practice factor and the accessibility factor are generic and consists of only a few items, but could easily be supplemented with additional items if the purpose require a broader assessment of these factors.

Respondent burden is a general concern in patient surveys, giving rise to a large amount of short forms, including short forms for measuring patient experiences in the primary doctor or outpatient setting.^34^ Our study showed that the PEQ-GP for chronic patients might be reduced from 20 items to a 7-item short form, consisting of five GP items and a single item for each of the other factors. The short form might be used in applications requiring shorter questionnaires, as it provides a uniquely efficient approach for brief yet comprehensive measurement. A previous study showed the adequacy of using single items to represent factors in short forms,^34^ but in contrast to that study we were not able to assess the reliability at the provider level. Thus, further research with adequate provider level samples should be conducted to evaluate reliability at the provider level.^9,34^

Global items like satisfaction and recommendation to others are normally separated from patient experience scales. In this study, we included all GP items in the analysis, also global items. We argue that this is justified because of the following reasons: (i) the definition of patient experiences of care used in this study is inclusive, also for global items; (ii) all candidate GP items include a direct assessment of the GP, and for chronic patients with long and frequent contact with their GP it makes sense to conceptualize a single latent factor; iii) all experience and global items falls well within the category of outcomes of health care from the patient perspective. Our study supported a single underlying GP factor, but results should be replicated in future research with even larger samples.

The response rate in the survey was 43%, which is a limitation for the current study. While the reported prevalence of diabetes among respondents in the total sample^33^ coincide with other studies,^35^ the prevalence of hypertension in the sample was relatively low.^36^ Furthermore, since we lack data about chronic conditions in the sample frame we were not able to assess the representativeness of the chronic respondent sample. Limitations of our evaluation of the psychometric performance include the lack of estimates for test–retest reliability and reliability estimates at the provider level, including for the single items in the newly developed short version. Further validation work related to different severity levels of chronic condition is also warranted, preferably based on larger samples than the current study. The current study used a conceptual and statistical approach to shorten the questionnaire, without direct input from patients about their priorities. We underline the importance of future research incorporating patients’ priorities, e.g. by asking a representative sample to rate the importance of each item,^37^ and then using this as an additional criteria when selecting items. The current study proposed a short form based on a survey with the long form, but there is no guarantee that the measurement properties will be reproduced when using the short form alone. Thus, we stress the importance of a separate validation study of the short form, in line with recommendations in the literature.^38^

As for other measures of PREMS, limitations are the long-term and external validity of the measure, given organizational and other societal changes and cross-national differences. To compensate, a robust assessment of the concordance between the purpose and context of measurement and the actual measure is a crucial starting point. Furthermore, validation work should be interpreted as a continuous process, not as a one-time procedure. For example, the choice of item 16 about organization of care for the short form is consistent with the Norwegian system as of 2021, given the results of our study. However, this decision may have been different for systems with stronger integration of other health professional groups, or in a future Norwegian system with stronger integration.

## Conclusions

The generic PEQ-GP is an adequate instrument for assessing patient experience with GP for persons with chronic conditions, with three empirically based scales covering evaluation of the GP, accessibility and practice. The instrument can be recommended for use in future applications in Norway and in other countries with similar organization and performance of general practice. The 7-item short form minimize respondent burden, but further validation work is warranted before large-scale use, including psychometric properties in separate, representative samples, patients’ priorities, and provider-level reliability.

## Supplementary Material

cmab133_suppl_Supplementary_Material_1

cmab133_suppl_Supplementary_Material_2

cmab133_suppl_Supplementary_Material_3

## Data Availability

The dataset is available from the corresponding author on reasonable request.
