# Supplementary figures and images for: Patient experiences with general practitioners: psychometric performance of the generic PEQ-GP instrument among patients with chronic conditions
Source: Fam Pract. 2021 Oct 20;39(3):519–26. doi: 10.1093/fampra/cmab133 (PMC9155158; doi:10.1093/fampra/cmab133)

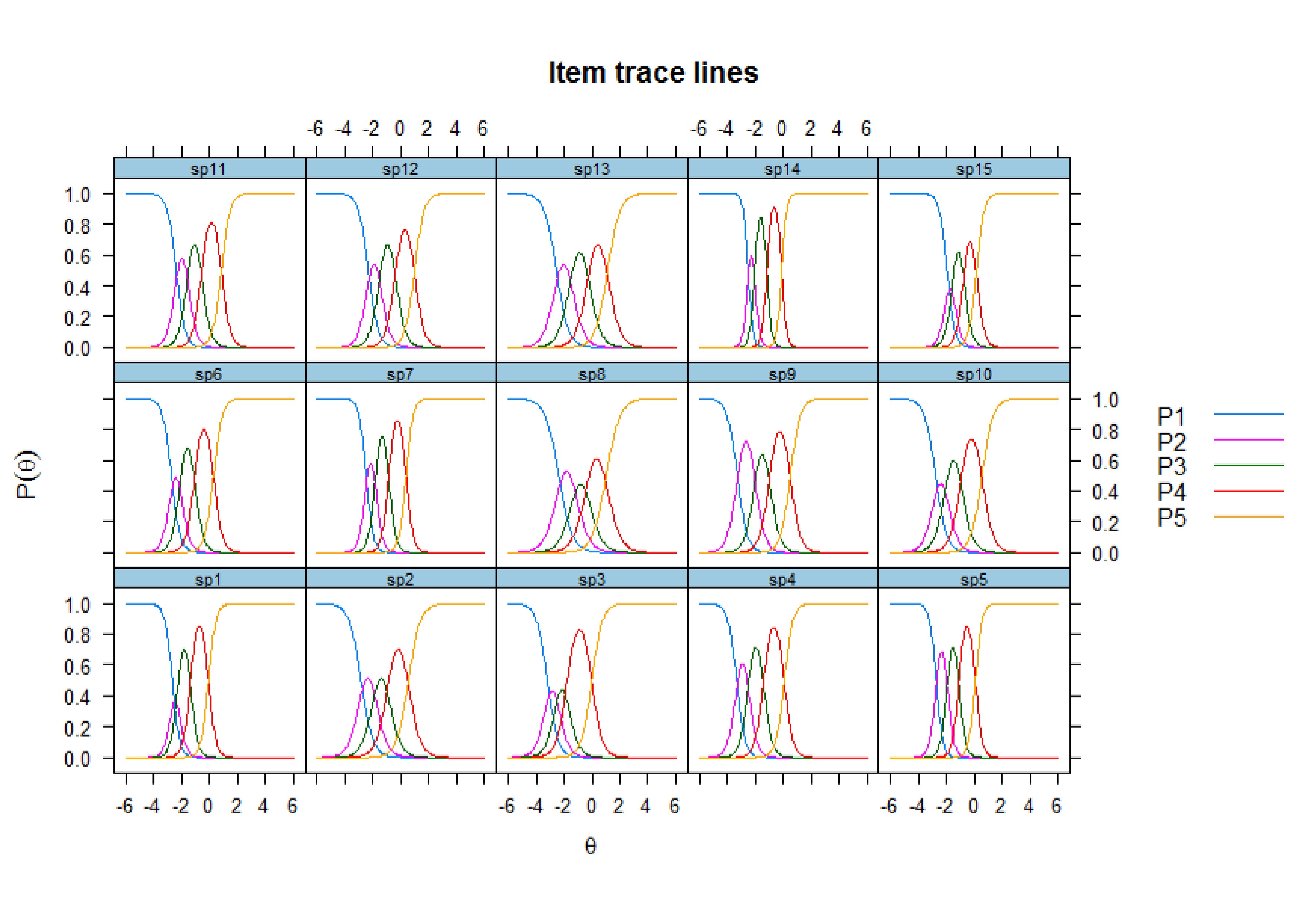

Supplement: cmab133_suppl_Supplementary_Material_1 [file cmab133_suppl_supplementary_material_1.jpeg]

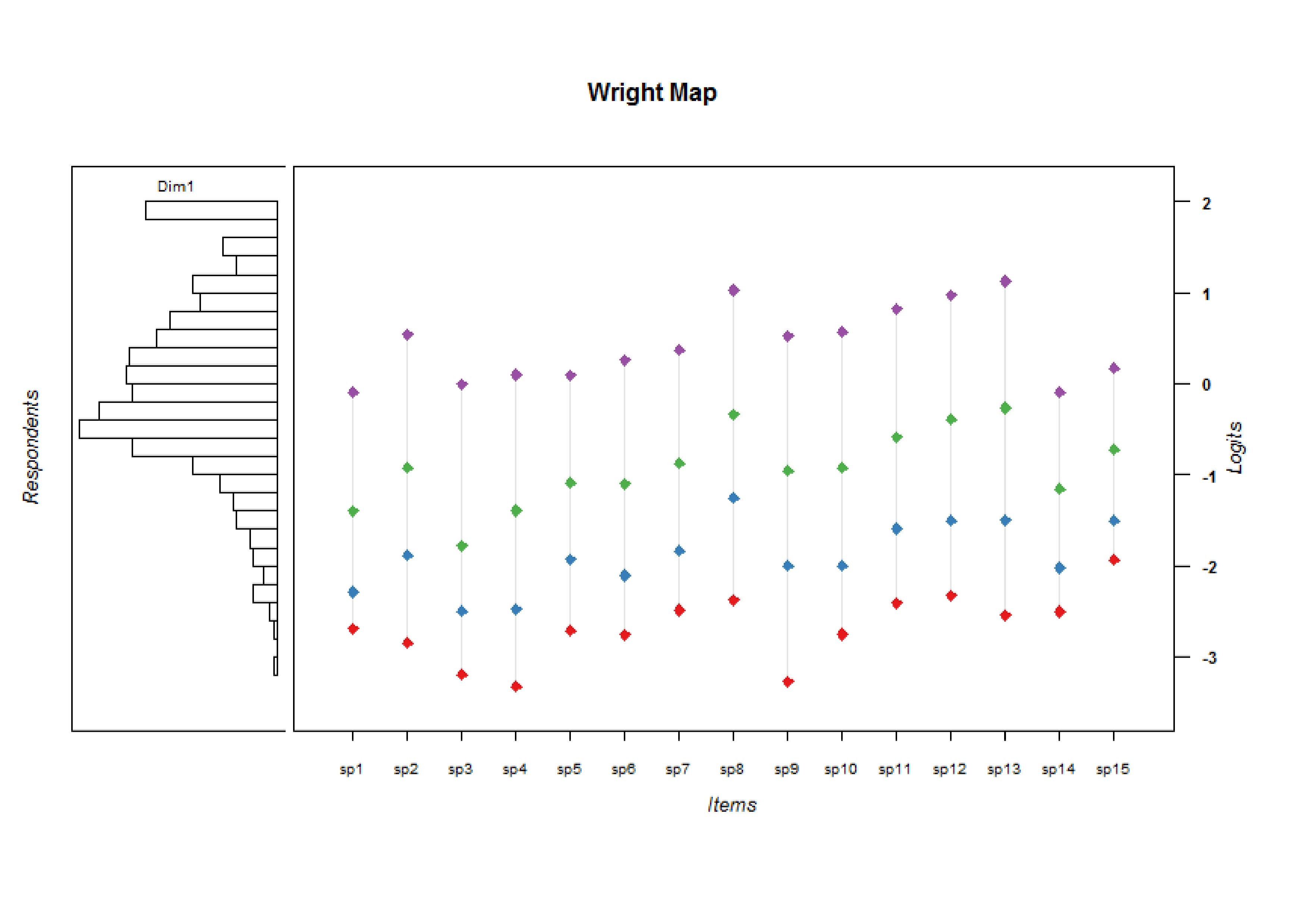

Supplement: cmab133_suppl_Supplementary_Material_2 [file cmab133_suppl_supplementary_material_2.jpeg]
